# Seeing the Value of Video: A Qualitative Study on Patient Preference for Using Video in a Veteran Affairs Telemental Health Program Evaluation

**DOI:** 10.1089/tmr.2021.0005

**Published:** 2021-05-31

**Authors:** Patricia V. Chen, Ashley Helm, Terri Fletcher, Miryam Wassef, Julianna Hogan, Amy Amspoker, Marylène Cloitre, Jan Lindsay

**Affiliations:** ^1^ Houston VA HSR&D Center for Innovations in Quality, Effectiveness and Safety, Michael E. DeBakey VA Medical Center, Houston, Texas, USA.; ^2^ Margaret M. and Albert B Alkek Department of Medicine, Baylor College of Medicine, Houston, Texas, USA.; ^3^ VA South Central Mental Illness Research, Education and Clinical Center, Houston, Texas, USA.; ^4^ Menninger Department of Psychiatry and Behavioral Sciences, Baylor College of Medicine, Houston, Texas, USA.; ^5^ VA National Center for PTSD, Menlo Park, California, USA.

**Keywords:** telehealth, video, mental health, patient preference, qualitative

## Abstract

**Background:** As the use of telemental health—mental health care delivered through video or phone—has increased in the era of COVID, it is important to understand patients' preferences and perspectives regarding the use of video for telehealth visits. A new web-based treatment program for veterans uses video visits with mental health experts to supplement its online cognitive behavioral therapy to treat clinically significant symptoms of depression and/or post-traumatic stress disorder.

**Objective:** As part of the program evaluation, Veterans were asked, “How important was it for you to be able to physically see your provider through video telehealth?” to understand whether they thought using video was important and why it may or may not be important.

**Materials and Methods:** The study uses data from the program's exit survey and exit interview. The surveys and interviews were conducted over a 19-month period. Surveys and interviews were conducted over the phone with note taking. Matrix and content analyses were used to analyze the qualitative data—predetermined themes and emergent themes were analyzed and inform findings.

**Results:** Seventy-three veterans completed a survey. Of these, 64 completed an interview. The majority of veterans surveyed (75%) said that it was “very important” to physically see their provider through video telehealth, 23% said that it was at least “somewhat important” or “not at all important.” This study highlights three main themes found in the qualitative data: patients discuss (1) advantages of using video, (2) why they dislike video, and (3) technological barriers to using video.

**Conclusions:** Being able to visually see a provider, and be seen by a provider, has distinct benefits for care and relationship building that are difficult to achieve over the phone. This has important implications for the future delivery of telemental health care and deserves consideration as patients and providers decide whether to use phone or video for remotely delivered care.

## Introduction

While telehealth has been available since the 1960s^1^ and deployed in a variety of health and treatment contexts,^2–4^ its use has surged across the country as the era of COVID has quickened telehealth's reach and adoption.^5,6^ Reflecting broader trends, the U.S. Department of Veteran Affairs (VA) has actively sought to promote and implement video telehealth since 2013.^7^ Before the pandemic, only 2% of mental health encounters within the VA health care system were through the VA's video telehealth to home platform, VA Video Connect. But by April 2020, with social distancing and stay-at-home orders in place, 90% of all VA mental health encounters were conducted remotely. A closer analysis of these VA telemental health encounters shows a mix of modalities being used—with a majority of care delivered through telephone. Within the VA health system, of the 90% of telemental health provided during April 2020, 65% was through phone.^8^

Thus, the growth in telemental health during the pandemic has largely involved patient encounters that take place over the phone. Lower barriers in terms of device and internet bandwidth requirements make phone more accessible for patients and providers alike. As clinics and providers work to continue to provide health services by any means possible, the importance of using video may be overshadowed by the impulse to connect quickly over the phone (even when video platforms are available).

However, clinical experts of telemental health and implementation encourage the use of video rather than phone, noting that use of video technology allows patients and providers to recreate social rhythms and can give additional context for care, such as allowing providers to see home environments, with patient permission.^9^ In mental health care settings, the use of video has been shown to have important implications for quality of care, enabling alliance building, presence, and improving patient–provider relationships. Furthermore, telemental health through video visits can confer additional advantages of convenience and accessibility,^10–12^ while *also* resulting in similar health outcomes to in-person care.^13,14^ Because telehealth will remain as part of the new normal for mental health care, even after the social distancing requirements are eased, it is an opportune time to explore whether and what advantages using video may bring.

This article is a qualitative study on patient perspectives toward the importance of using video for telemental health. While it does not explicitly compare video and phone, it provides an exploratory foundation to begin this important conversation, presenting the salient themes on the advantages of video for remote mental health care. While earlier studies show similar ratings and comparable effectiveness between outcomes,^15,16^ this study uses qualitative methods to understand *whether* using video during telehealth is important to patients, and if so, *why* using video may or may not be important to them.

## Materials and Methods

### Evaluating a VA telemental healthcare program

Data used in this study were collected to evaluate a mental health care program for veterans (which continues at the time of writing). The program is designed for veterans who have experienced trauma, particularly military sexual trauma, and have symptoms of post-traumatic stress disorder (PTSD) and/or depression as indicated by a positive screen on either the Primary Care PTSD Screen for DSM-5 (PC-PTSD-5)^17^ or the 2-item Patient Health Questionnaire (PHQ-2),^18^ respectively. Program content focuses on greater emotional awareness and emotional regulation, and relationship management. Most veterans who participated did so upon recommendation from their providers. A notable feature of the program is its hybrid approach to learning and care: participants work through self-guided modules while also meeting with a professionally trained therapist who is assigned to them for the duration of the program. While providers are all clinically licensed therapists working for the VA, they are presented to veterans as “coaches” as their primary role in this program is to give veterans the opportunity to review topics, seek clarity, and receive additional guidance on modules' content.

This program lasts 10–15 weeks for veterans. The online web content consists of 10 self-guided modules. Meetings with coaches, typically held after a veteran has worked through a module, are conducted through a video platform for telehealth and last 50 min. Veterans can meet their coaches either 5 times (meeting after every two modules) or 10 times (meeting after each module). Veterans are connected to their therapist through a video telehealth platform—never in person—and only use telephone when technological difficulties make the use of video untenable. Coaches are instructed to use the video platform as the default modality for all sessions and to use phone only if necessary.

As part of evaluation processes for the program, five members from the research team (coauthors A.H., M.W. included) took turns to conduct exit surveys and exit interviews with patients. The exit survey used 23 structured questions; one of which asked patients to rate how important it was to physically see their provider through video telehealth during the program. The exit interview (conducted immediately after the survey) used 10 open-ended questions to further probe patients' previous experience with remotely delivered mental health care, if any, why and how they got involved in the program, more details about their experience with self-guided portions of the program, and experiences with their therapist-coach.

### Participants

Program participants were U.S. veterans enrolled in nine VA facilities across the country. From September 2018 to March 2020, 202 participants completed the program. Upon completion of the program, a coauthor (M.W.) requested permission from the program's therapists to invite patients to participate in the exit survey and interview. If patients did not complete their last session with their therapists or if permission was not given, patients were not contacted. For those eligible for an exit survey and interview, a letter was sent to them communicating the purpose of the survey and interview and allowing them to opt out if desired. Patients were called at least three times. Seventy-three (>30% response rate) respondents agreed to participate in the exit survey.

### Data

This study draws from data collected through exit surveys and interviews. Survey and interview protocols were reviewed by PI's affiliated institutions' IRB, designated as Quality Improvement, and determined to be exempt from IRB review. Participating respondents were informed that their participation was confidential and voluntary before the start of the survey and interview. While the research team used two separate instruments for the survey and interview, interview questions immediately followed survey questions on the same phone call with the respondent. Survey and interviews were 1:1 and lasted ∼45–60 min.

Survey and interviews were conducted over secured phone lines at a research center. They were not audio-recorded. Rather, while one person conducted the interview, another member of the research team took detailed notes (including coauthors, A.H. and M.W.). Data collection took place from 2018 to 2020, before March 2020 when the COVID-19 pandemic began in the United States.

This study draws from the survey questions asking “How important was it for you to be able to physically see your provider through video telehealth?” with options to rate along a three-pt scale (1: not at all important, 2: somewhat important, and 3: very important). This question was followed by the open-ended question, “What did you like about working with the therapist over video telehealth?” (Supplementary Appendix SA1). Notably, the exit interview did not explicitly ask patients to compare video with phone. However, many respondents made the comparison on their own, further explaining how they felt the quality of their care and engagement with their provider would have been different if it had been over the phone. Responses to these questions constitute the core qualitative data used in this study.

The study also draws from responses to the interview questions. The interview used the following open-ended questions: *What kind of technology have you used to talk with a mental health provider? What do you like or dislike about these different modes of delivery? How comfortable are you with technology? Now that you've completed the program, have your thoughts changed at all about using web and video technology for your mental health care? If yes, how so?* (Supplementary Appendix SA1). Any relevant responses to using video relative to other modalities were included in the analysis.

### Qualitative analysis

Analysis for this study used strategies and best practices from matrix analysis^19^ and qualitative content analysis.^20,21^ Our analysis took the following process: (1) Responses to the question on video preferences and all relevant text (any time a patient discussed preferences and attitudes toward telehealth modalities) from the interview records were extracted from the original record files and compiled into a matrix. (2) Responses were reviewed several times to build familiarity with content. (3) The first author (P.C.) used an inductive approach that allowed themes to emerge through the review process and through reorganizing and grouping of quotes that spoke to similar topics and issues. (4) The second author (A.H.) independently reviewed the emergent themes and quotes to mitigate bias and provide an interpretive check on groupings. (5) The authors discussed the themes and groupings, coming to a consensus on any disagreements. (6) The themes were further defined and explicated to describe why using video may or may not be important to the patient.

## Results

Seventy-three veterans participated in the survey. Of these, 64 agreed to provide additional information through interviews, with 9 opting out of the interview due to time constraints. The demographic table below details proportions on gender, race and ethnicity, age, relationship status, educational level, and employment (Table 1).

**Table 1.** Veteran Demographic Characteristics (*n* = 73)

Gender (%)		Education level (%)	
Male	42.4	Some high school	1.3
Female	56.2	Completed high school	10.9
Transgender	1.3	Some college	43.8
		Completed college	32.8
Race/ethnicity (%)		Postgraduate	10.9
American Indian or Alaskan Native	2.7		
Asian	0.0	Employment status (%)	
Black or African American	10.9	Full-time	32.8
Native Hawaiian or Pacific Islander	0.0	Part-time	13.6
Hispanic, Latino(a), or Mexican American	6.8	Not working	21.9
White or Caucasian	65.7	Retired	31.5
Middle Eastern or North African	0.0		
Two or more	13.6	Rural/urban (%)	
		Rural	36.9
Age		Urban	58.9
Average age (years)	46		
Age range	25–78	Evidence-based practice within the last year (%)^a^	
		Yes	6.8
Relationship status (%)		No	93.1
Married	61.6		
Single	16.4		
Divorced	20.5		
Widowed	1.3		
Caregiver (%)^b^			
Yes	39.7		
No	60.3		

^a^ Veteran was treated with an evidence-based practice during 2018–2019.

^b^ The Veteran had child or eldercare duties.

Of the 73 respondents participating in the program's exit survey, 75% (55) said that it was “very important” to physically see their provider through video telehealth. Fifteen percent (11) said that it was at least “somewhat important.” Only 8% (6) of respondents said that it was not at all important, and 1 respondent did not provide an answer. Of the 64 who responded to interview questions on whether they had had any previous experience with telehealth, 53% (34) said that they had never had any experience, and 46% (30) had ∼1 encounter through telehealth before the start of the program.

### Theme one: patients discuss advantages of using video

Patients' responses to why it is important to see their provider through video coalesced around three subthemes: (1) enhanced communication, (2) more accountability, and (3) better patient–provider relationship.

#### Enhanced communication

Patients described how expressions, body language, and mannerisms were extremely important to them. Some explicitly noted that this would not have been possible with a phone conversation: “It's better than talking on the phone. Seems ineffective to do over the phone. [You] Get to see facial expressions, body language. It's extremely important, non-verbal communication” (57-year-old white male). Another enumerated the importance of nonverbal cues: “75% of communication is nonverbal. Good to see expression on people's faces. It adds another layer to communication that phone couldn't have” (36-year-old white female).

#### More accountability

Several patients also said that connecting with their provider through video gave an added sense that they were “working,” and that there was more “accountability.” “When you can see the person, it's much more serious. Human connection sets the tone. Creates the atmosphere of working on something” (44-year-old white male). Another patient articulated, “We had to use the phone one time, next time I really appreciated video. Contact formalized with appointment, accountability to adhere to that time and be there” (72-year-old, American Indian male). Nonverbal cues seen by their providers also lent themselves to creating a greater sense of accountability: “Being able to see a person and talk to them is more accountability. She could pick up on things and notice things” (35-year-old Black female).

#### Better patient–provider relationship

Patients also described increased connection with their provider: “Felt more of a connection with her, felt like she really cared and was really listening. Weren't in the same room but she could see my reactions and vice versa. Better than just being on the phone” (37-year-old Hispanic/Latino female).

Using video allowed a better relationship because it “establish[ed] more of a personal connection” (32-year-old multiracial male) and helped to “establish trust” (35-year-old American Indian female). Having a visual on their provider allowed patients to see that their provider “really cared and was really listening” (37-year-old Hispanic/Latino female). One patient emphasized that this would not have been possible over the phone, and said that with video, “It feels more like you're with someone face-to-face. You feel more connected. Did one session by phone, didn't feel the same. It was helpful to have face to face” (47-year-old white male).

### Theme two: patients discuss why they dislike using video

Not all patients were enthusiastic about using video for telemental health. Twenty-three percent (17) said that using video was either “somewhat important” or “not at all important.” Among the six who said that video was not at all important, three explicitly said that they would have been fine with using a telephone. However, even some patients who rated video as “very important” discussed frustrations with video. Interviews revealed three main reasons why video was not preferable for some patients: being on video felt awkward; they had privacy concerns; or they had a preference for in-person care.

#### Feeling awkward

The majority of veterans who disliked video cited their own discomfort with this modality: “I still prefer in person because it's less awkward than the webcam for me… Video is not necessarily appealing—[there are] delays, talking over each other… I wasn't that comfortable over video. It was awkward. I don't like being on video or on camera; it makes me anxious” (35-year-old white female).

#### Privacy concerns

Three respondents had privacy concerns, particularly in the context of having family or others nearby listen to confidential conversations, and as one put it “The main thing is when you're doing a video is that when there are other people in the house, you don't want to share it with them because it's private and you're wondering if the neighbors are hearing it. You know, it blocks my ability to be as honest. Paranoid that others can hear me” (57-year-old mixed race female).

#### Prefer in person

Four veterans said explicitly that they preferred in-person care. Interestingly, two of those who said that having a video was “very important” still preferred in-person care and would have had their sessions in-person if it were more logistically convenient: “In person is a lot better, but for the distance, telehealth is a good thing. [Telehealth]just don't have that personality connection. [There is]not as much personal involvement” (52-year-old white male).

### Theme three: patients discuss technological difficulties with using video

Regardless of whether they liked using a video platform for their care, most participants (77%) reported at least some technological issues with using video.

One of the technological issues patients faced was poor video quality: “Video quality was sporadic … Sometimes I could see her, she couldn't see me” (52-year-old white male). Some patients described connectivity issues that kept them from accessing the video platform: “It was just difficult. I'd get the email, click on the appointment, and wouldn't connect” (59-year-old white male). And for those less experienced with technology, learning to use the video platform was an additional challenge: “It took me a long time getting used to the tablet. It was a little intimidating trying to learn how to work with the tablet” (68-year-old white male).

Technological problems were the primary barriers to using video telehealth and often drove patients and providers to use their phone. As one put it simply, “[The video] didn't work sometimes. We had to redirect about 2 or 3 times. Not sure if it was our end or the VA. The telephone was a lot easier than the video” (59-year-old white male).

Despite frequent issues with technology, these problems did not actively prevent completion of the program. Most participants were willing to troubleshoot through technology glitches and continue with sessions.

## Discussion

Telemental health—the delivery of mental health care through video platforms or over the phone—has taken on a new salience in the era of COVID-19. In a time of great uncertainty and restructuring of societal norms, it has allowed clinics to adhere to safety precautions and continue care. Even before the need for social distancing, telehealth's value for reaching those in rural areas,^22,23^ for enabling access to care during weather-related disasters,^24,25^ and for providing patients with trauma and anxiety disorders the ability to connect from the comfort of their homes^26,27^ made a clear case for expanding its use. Now, as we begin to settle into the new norm in which telemental health is widespread, it is important to consider how different modalities can attenuate the patient experience.

This study considers patient preferences on whether video visits are important for telemental health and explores why using video matters to patients. A strong majority of patients said that it was important to be able to see their provider; being able to see their provider allowed better communication, primarily because it allowed both patient and provider to use nonverbal cues. Nods of empathy and encouragement, positive or negative facial expressions—these cues helped facilitate interpretations and intention. Furthermore, having a visual component during telecommunication requires parties to adhere to more social norms similar to those required for in-person meetings, such as focusing on the meeting (rather than attempting to multitask as one might over the phone). Both patient and provider thus need to be more present during sessions. Patients sensed and appreciated that their providers were more present and attentive, and that they could see this from them.^28,29^ Enhanced communication and increased accountability through a video platform worked in concert to build a strong relationship with their provider, which was foundational to engagement and retention in the program.^30–32^

### Limitations

The study had some limitations. First, the data were collected as part of a larger project to evaluate the implementation of a program, not for the sole purpose of comparing telemental health modalities. Furthermore, no direct comparison between patients who used phone versus those who used video could be made—all participants used video and were only asked about their experience with the use of video during the program. Second, there may be some selection bias among those who agreed to participate in the exit interview, with those having more positive experiences being more willing to provide feedback. Finally, the patient experience here speaks primarily to the use of telemental health in a single program setting, and we can only tenuously consider how key findings may be applicable to other mental health programs and medical settings.

## Conclusion

As an exploratory evaluation of patient preferences for telemental health modalities, it is clear that most patients think it is important to use video. Being able to visually see a provider, and be seen *by* a provider, has distinct benefits for care and relationship building. To the extent that a positive, communicative, and trusting relationship with a provider has real bearings on health outcomes for patients, the use of video can enable more effective care while allowing patients to overcome logistical, access, and safety barriers associated with in-person care. Future studies may further explore and systematically test for differences between video and phone platforms or explain how the use of a video platform may vary in different medical contexts.

## Supplementary Material

Supplemental data

## Supplementary Material

Supplementary Appendix SA1

## Abbreviations Used

PTSD: post-traumatic stress disorder
VA: Veteran Affairs
