# Supplementary material for: Seeing the Value of Video: A Qualitative Study on Patient Preference for Using Video in a Veteran Affairs Telemental Health Program Evaluation
Source: Telemed Rep. 2021 May 31;2(1):156–62. doi: 10.1089/tmr.2021.0005 (PMC8812285; doi:10.1089/tmr.2021.0005)
Supplement: Supplemental data [file Supp_AppendixSA1.docx]

EXIT SURVEY

WebSTAIR Coach5/Coach10

POST-TREATMENT

We are very interested in learning about your experiences with the webSTAIR online program. We would like to ask you a few questions to get any feedback that you would like to provide. Please feel free to be as honest as possible so that we can make any changes to improve our program. We encourage you to be open about what worked and didn’t work for you.

***(complete the next section prior to starting interview)***

As a reminder….

We show that you started the webSTAIR program on ______________ (date of first session).

Your webSTAIR coach was ___________ (coach name).

We show that you completed _________ number of modules and had ________ (total number) coaching sessions

Your last session occurred on _______________.

The following questions are about your experiences with the webSTAIR program. Do you have any questions before we get started?

1. What did you like about the webSTAIR program?

*___________________________________________________________________________*

*___________________________________________________________________________*

*___________________________________________________________________________*

1. What didn’t you like about the program?

*___________________________________________________________________________*

*___________________________________________________________________________*

*___________________________________________________________________________*

1. What suggestions do you have for changes or improvements to the webSTAIR program?

*___________________________________________________________________________*

*___________________________________________________________________________*

*___________________________________________________________________________*

1. What, if any, impact did the program have on your life?

*___________________________________________________________________________*

*___________________________________________________________________________*

*___________________________________________________________________________*

1. How helpful was each of the following components of the webSTAIR website?

Not at all helpful 1

Somewhat helpful 2

Very helpful 3

- Use of the toolbox
- Skills practice
- Wellness check
- Positive self-statement
- Course list (access to all modules)
- Achievements feature (i.e. badges)

b. What did you like best about the webSTAIR website?

c. What did you like least about the webSTAIR website?

1. I’d like to ask you about the modules you completed. Please rank the two most helpful modules and the two least helpful.

____Module 1 – Emotional Awareness

____Module 2 – Managing Emotions: Focus on Body

____Module 3 – Managing Emotions: Focus on Thoughts

____Module 4 – Managing Emotions: Focus on Behaviors

____Module 5 – Tolerating Distress to Reach Valued Goals

____Module 6 – Understanding Relationship Patterns

____Module 7 – Identifying your Relationship Patterns

____Module 8 – Changing Relationship Patterns – Focus on Assertiveness and Closeness

____Module 9 – Increasing Flexibility and Compassion for Self and Others

b. What did you like about module___ (#1 ranking)?

c. What did you like about module___ (#2 ranking)?

d. Why did you rate module___ (#9 ranking) as less helpful?

e. Why did you rate module___ (#8 ranking) as less helpful?

1. Overall, did the webSTAIR program meet your needs?

Met none of my needs 1

Met some of my needs 2

Met most of my needs 3

Met all of my needs 4

1. Now that you have completed the webSTAIR program, do you plan to seek additional mental health services? (If yes) What kind of services do you think would be helpful?

*___________________________________________________________________________*

*___________________________________________________________________________*

*___________________________________________________________________________*

1. We show that you completed ________ (total number) modules on the webSTAIR website. Was this the right number of modules for you? Would you have preferred more modules or fewer modules?

Right number of modules for me 1

Would have preferred fewer modules 2

Would have preferred more modules 3

Why?

*___________________________________________________________________________*

*___________________________________________________________________________*

*___________________________________________________________________________*

1. We show that you had ________ (total number) sessions with ________ (therapist name). Was this the right number of sessions for you? Would you have preferred more sessions or fewer sessions?

Right number of sessions for me 1

Would have preferred fewer sessions 2

Would have preferred more sessions 3

Why?

*___________________________________________________________________________*

*___________________________________________________________________________*

*___________________________________________________________________________*

1. The webSTAIR program provided you with the opportunity to work with a therapist over video telehealth. How important was it for you to:

Be able to physically see your provider through video telehealth?

Not at all important 1

Somewhat important 2

Very important 3

1. What did you like about working with the therapist over video telehealth?
2. Do you recall your coach prompting you to complete modules between sessions either by phone or through messages through MyHealthEVet?

Yes 1

No 0

a. How did you prefer to receive those prompts?

Phone calls 1

MyHealthEVet 2

1. Did you contact your coach between sessions?

Yes 1

No 0

- 1. If yes, how did you contact your coach?

Phone call 1

MyHealthEVet 2

- 1. What were the reasons you contacted your coach? (select all that apply)

Rescheduling a session

Password

Technology issues (webSTAIR)

Technology issues (video-to-home)

Problems understanding a skill

Other

1. How important was it for you to be able to talk to your therapist between coaching sessions?

Not at all important 1

Somewhat important 2

Very important 3

1. Did you experience any difficulties with the video connection to ________ (coach name)?

Yes 1

No 0

b. If yes, what were the difficulties you experienced?

1. Did you experience any difficulties with the technology for the webSTAIR website?

Yes 1

No 0

b. If yes, what were the difficulties you experienced?

1. What, if any, factors made your participation in the webSTAIR program difficult? (e.g., technology difficulties, difficulty scheduling sessions, family issues, health issues, # of people you had to talk to get started with webSTAIR, etc).

*___________________________________________________________________________*

*___________________________________________________________________________*

*___________________________________________________________________________*

1. Some Veterans have reported challenges to receiving mental health care in person at the VA or clinics for a variety of reasons. Have any of the following made it difficult for you to seek mental health care in person at the VA?
2. Lack of transportation
3. Distance/travel time to VA or clinic
4. Taking time off from work or school
5. Arranging childcare
6. Don’t feel comfortable at VA or clinic
7. Don’t want others to know you are seeking MH care
8. Don’t feel you can be open and honest at VA or clinic
9. How likely are you to try another web-based therapy program with a coach?

Not at all likely 1

Somewhat likely 2

Very likely 3

1. How likely are you to try another web-based therapy program without a coach?

Not at all likely 1

Somewhat likely 2

Very likely 3

1. How likely are you to see a MH provider through video telehealth in the future??***

Not at all likely 1

Somewhat likely 2

Very likely 3

1. How likely are you to see a medical provider through video telehealth in the future?

Not at all likely 1

Somewhat likely 2

Very likely 3

1. If somewhat or very likely, for what type(s) of care?
2. Any additional comments?

EXIT INTERVIEW

WebSTAIR Coach5/Coach10

POST-TREATMENT

***[Interviewer instructions: verify patient identity]***

***[Interviewer instructions: please read the following to the patient]*** *We’d like to know some more about your experience completing the video-to-home webSTAIR program. Your feedback will help us better serve your fellow Veterans.*

*Our conversation should last about 30 minutes or so. Just a reminder that everything you say to me today is confidential. There is no way to link anything you say to me back to you.*

*If there are any questions you’re not comfortable answering, just let me know. Also, you can stop the interview at any time.*

*I’ll be taking some notes today while we talk. Again, your identity will be protected and any information that can identify you will be removed from the feedback you provide to me today.*

*These are very open-ended questions so I encourage you to provide as much detail as you would like.*

**To start off, I’d like to ask you some questions about your previous healthcare experiences**

1. Had you ever talked with a mental health provider such as a psychologist, psychiatrist, social worker, or addiction therapist at the VA prior to webSTAIR? (Follow-up: talked with mental health providers outside of VA)
   1. (if yes) Can you describe your overall experience(s)?
      1. *clinical probe*: duration and focus of treatment, setting/context, rapport with provider, likes/dislikes of treatment
      2. *technology probe*: kind of technology used, likes/dislikes with mode of delivery, comfort level with technology

**Now I’d like to know more about how you got started with the webSTAIR program.**

1. How did you hear about the webSTAIR program? Who explained the program to you? How was the program explained to you?
   1. *Clinical probe*: How was the coaching component explained? (probe: treatment content, role of coach, Veteran’s understanding of how webSTAIR is different from traditional psychotherapy)
   2. *Technology probe*: How was the web component explained? How was the video component explained? (probe: what was said, demeanor/attitude of provider who introduced web-based program and video delivery format)
      1. Now that you’ve completed the program, have your thoughts changed at all about using web and video technology for your mental health care? (If yes) How so?
   3. Marketing probe: Did you receive a brochure about webSTAIR prior to starting? If so, did it have any impact on your decision to enroll in webSTAIR?
2. What are the main reasons you decided to participate in webSTAIR?
   1. *Clinical probe*: hoping to address specific symptoms/problems, skill based vs. trauma-focused, coach support
   2. *Technology probe*: convenience, flexible schedule, logistical barriers, stigma, access to care, previous experiences with in-person mental health care at the VA, previous experience with telehealth

**We talked about some of this in the earlier exit interview, but we’d like to learn just a little bit more about your experiences with the webSTAIR program.**

1. In the earlier exit interview, you mentioned that module X and Y were the most helpful to you. Can you provide some specific examples how these modules helped you?
2. Which between-session tasks, worksheets, or practice did you do?
   1. Probe: How did you complete these (online in webSTAIR program/tools section; through printed handouts/worksheets; on your own (without handouts or other supports); with assistance of webSTAIR coach)
   2. Which of these were the most helpful?
3. What was it like working through the modules on your own without the assistance of your coach?
   1. What was it like going through several modules before discussing with your coach?

**Now I’d like to know more about your experiences with your coach.**

1. What was your relationship with your coach like? (probe for specific examples, likes/dislikes, provider rapport) How important do you think the relationship with your coach was to working through the webSTAIR online program? (probe reasons)
   1. What impact did your coach have on your understanding of the skills?
   2. What was your comfort level sharing information with your coach over video about your life, thoughts and feelings as you worked through the webSTAIR program?

**Thank you for sharing your feedback with me today! I just have a few more questions.**

1. Did you consider stopping webSTAIR at any point? Why?

If *yes*: What influenced your decision to stay in treatment/discontinue?

1. Would you recommend this program to other Veterans? Why or why not?
2. Is there anything else that you’d like to share with us?

**Thank you for your time!**
